# Hotspots of female genital mutilation/cutting and associated factors among girls in Ethiopia: a spatial and multilevel analysis

**DOI:** 10.1186/s12889-021-10235-8

**Published:** 2021-01-21

**Authors:** Tesfahun Taddege Geremew, Muluken Azage, Endalkachew Worku Mengesha

**Affiliations:** 1 Public Health Emergency Management (PHEM) Directorate, Amhara Public Health Institute (APHI), Bahir Dar, Ethiopia; 2 School of Public Health, College of Medicine and Health Sciences, Bahir Dar University, Bahir Dar, Ethiopia

**Keywords:** Genital mutilation/cutting, Circumcision, Spatial, Multilevel

## Abstract

**Background:**

Female genital mutilation/cutting (FGM/C) is a harmful traditional practice that violates the human rights of girls and women. It is widely practiced mainly in Africa including Ethiopia. There are a number of studies on the prevalence of FGM/C in Ethiopia. However, little has been devoted to its spatial epidemiology and associated factors. Hence, this study aimed to explore the spatial pattern and factors affecting FGM/C among girls in Ethiopia.

**Methods:**

A further analysis of the 2016 Ethiopia Demographic and Health Survey data was conducted, and a total of 6985 girls nested in 603 enumeration areas were included in this analysis. Global Moran’s I statistic was employed to test the spatial autocorrelation, and Getis-Ord Gi* as well as Kulldorff’s spatial scan statistics were used to detect spatial clusters of FGM/C. Multilevel logistic regression models were fitted to identify individual and community level factors affecting FGM/C.

**Results:**

Spatial clustering of FGM/C was observed (Moran’s I = 0.31, *p*-value < 0.01), and eight significant clusters of FGM/C (hotspots) were detected. The most likely primary SaTScan cluster was detected in the neighborhood areas of Amhara, Afar, Tigray and Oromia regions (LLR = 279.0, *p* < 0.01), the secondary cluster in Tigray region (LLR = 67.3, p < 0.01), and the third cluster in Somali region (LLR = 55.5, *P* < 0.01). In the final best fit model, about 83% variation in the odds of FGM/C was attributed to both individual and community level factors. At individual level, older maternal age, higher number of living children, maternal circumcision, perceived beliefs as FGM/C are required by religion, and supporting the continuation of FGM/C practice were factors to increase the odds of FGM/C, whereas, secondary or higher maternal education, better household wealth, and regular media exposure were factors decreasing the odds of FGM/C. Place of residency, Region and Ethnicity were also among the community level factors associated with FGM/C.

**Conclusions:**

In this study, spatial clustering of FGM/C among girls was observed in Ethiopia, and FGM/C hotspots were detected in Afar, Amhara, Tigray, Benishangul Gumuz, Oromia, SNNPR and Somali regions including Dire Dawa Town. Both individual and community level factors play a significant role in the practice of FGM/C. Hence, FGM/C hotspots require priority interventions, and it is also better if the targeted interventions consider both individual and community level factors.

## Background

Female genital mutilation/Cutting (FGM/C), also known as “female circumcision”, refers to “all procedures involving partial or total removal of the external female genitalia or other injury to the female genital organs for non-medical reasons” [1]. It is a violation of girls’ and women’s right to life, right to physical integrity, and right to health, as it damages healthy genital tissue and can lead to severe consequences for girls’ and women’s physical and mental health [2, 3].

FGM/C is associated with adverse health consequences in women over both the short and long terms [1, 4]. Immediate complications include severe pain, excessive bleeding, shock, difficulty in passing urine, delayed or incomplete healing, and infections, whereas, long-term consequences can include chronic pain, decreased sexual enjoyment, sterility, recurring urinary tract infections, the formation of deride cysts, birth complications, and psychological consequences, such as post-traumatic stress disorder [1, 4, 5].

Global estimates indicated that more than 200 million women and girls have experienced FGM/C [6, 7]. In Africa, the prevalence of FGM/C is still highly prevalent despite its decline in the last three decades. The prevalence decreased from 57.7% in 1990 to 14.1% in 2015 in North Africa, from 73.6% in 1996 to 25.4% in 2017 West Africa, and from 71.4% in 1995 to 8.0% in 2016 in East Africa [8]. In Ethiopia, the prevalence of FGM/C among girls declined from 24% in 2005 to 16% in 2016 [9, 10].

Despite international and national efforts to eliminate the practice of FGM/C, it has been widely practiced mainly in Africa for various reasons [1, 11]. Reasons that parents give for the procedure include preserving chastity, ensuring marriageability, rite of passage, improving fertility, religious requirement, and enhancing sexual pleasure for men [3]. Also, the practice is often presented as part of a girl’s initiation into womanhood within her own particular community, and as a way of controlling women’s sexuality [12].

The government of Ethiopia has committed to eliminating the practice of FGM/C by 2025 [13]. However, it is still widely practiced across the communities (16% among girls aged 0–14 years and 65% among women aged 15–49 years) [10], and both individual level and community level factors may play an important role in the continuation the practice.

Study findings on FGM/C in Ethiopia have focused solely on prevalence, characteristics of individuals affected and individual level factors associated with FGM/C [14–19]. The findings of such studies are insufficient and fail to capture the spatial epidemiology and community level factors affecting the practice of FGM/C beyond individual level factors.

In this study, Kulldorff’s spatial scan statistic was used since it is widely suggested in detecting appropriate local clusters compared to other spatial analysis techniques [20–22], mainly for two reasons. First, SaTScan can detect a cluster of any size between zero and a maximum limit defined by the user. Second, spatial scan statistic has a higher power of detecting local clusters than other available methods [21, 23]. However, this scan statistic also has limitations: it uses a circular window to define the potential cluster areas and thus has difficulty in correctly detecting actual noncircular clusters. In addition, SaTScan is sensitive to user controlled parameter choices (e.g., the maximum circle size, defined as the percentage of the total population at risk), and produces less usable clusters when inappropriately choosing parameters (i.e., heterogeneous contents). Thus, this study employed both spatial statistical techniques to overcome such problems.

In addition, previous studies identified individual level factors associated with FGM/C using a basic ordinary logistic regression model that has less statistical power, and could not control the nesting effect of clusters at different levels [14, 16, 18, 19, 24]. Thus, it is better to apply appropriate statistical methods for a more comprehensive and sound analysis to consider the cluster effect using multilevel analysis. Therefore, the study aimed to explore the spatial pattern of FGM/C and associated factors among girls in Ethiopia using a SaTScan spatial and multilevel analysis.

## Methods

### Study settings

This study was conducted using the data from the 2016 Ethiopia demographic and health survey (EDHS). In Ethiopia, this national and subnational representative household survey is conducted every five years. Being the most populous country in the region, Ethiopia is located in the horn of Africa between 3 and 15 degrees north latitude and 33 and 48 degrees east longitude [25]. Ethiopia is administratively divided into regional states and chartered zones, districts (woreda) and kebeles (the smallest administrative units). For the 2007 Ethiopian population and housing census (PHC), the kebeles were sub-divided into enumeration areas (EAs), which were used as a primary sampling unit for the 2016 EDHS [10].

### Study design and period

A cross-sectional data analysis was conducted using the secondary data from the 2016 EDHS, which was collected from January 18 to June 27, 2016.

### Study population and eligibility

The study population of this study were all girls aged 0–14 years in the randomly selected enumeration areas (EAs) of Ethiopia. Girls from the EAs with no geographical coordinates were excluded from this study.

### Sampling technique and sample size

In the 2016 EDHS, data were collected through a stratified, two-stage cluster sampling technique using the complete list EAs for the 2007 PHC as a sampling frame. The primary and secondary sampling units were EAs and households, respectively.

The 2007 PHC had a complete list of 84,915 EAs, and from this sampling frame a total of 645 EAs (202 urban and 443 rural) were selected with probability proportional to EAs size (PPS). In the second stage, 28 households per cluster were selected with an equal probability systematic selection. Overall, 16,650 households were interviewed successfully in this survey, yielding 98% household response rate. From those interviewed households, 16,583 women in the reproductive age were identified for individual interviews, and 15,683 women were interviewed successfully, yielding a response rate of 95%. Among the household in which the 15,683 women interviewed, 7795 households were selected for FGM/C interviews. By excluding 321 girls from the EAs with no geographic coordinates, 6985 girls aged 0–14 years were included for this study. The detailed methodology has been published in the 2016 EDHS final report [10].

### Data source and extraction

The datasets used for this study are publicly available in the DHS Program repository (https://www.dhsprogram.com/data/dataset_admin) to all registered users, downloaded with permission. After reviewing and understanding the details of the EDHS data structure and dataset types, we selected the recommended dataset type for girls’ circumcision. Data on FGM/C, and its potential explanatory variables (individual and community level variables) were extracted.

### Study variables and measurement

The outcome variable of this study is female genital mutilation/cutting status, and it was measured by asking women who had daughters whether any of their daughters born in 1992 or later had been circumcised. If a girl had been circumcised, the FGM/C status was classified as “Yes”, otherwise “No”.

### Explanatory variables

As described in Table 1, both individual and community level variables were included as potential explanatory variables of daughter’s FGM/C. Individual-level (level I) variables include women’s and their daughters’ background characteristics, while community-level (level II) variables include the common characteristics of study subjects based on the enumeration areas or clusters within the 2016 EDHS data. Some community level variables-i.e., proportion of community with regular media exposure, proportion of community in the poor wealth quantile, and women’s literacy rate were generated by aggregating the individual level data into clusters. Using the proportion of each variable aggregated per cluster, the variables were recoded into “high” and “low” groups based on the national median values. Median values were used to categorize the community level variables as high and low since the aggregated values were not distributed normally (Table 1).

**Table 1:** Independent variables and categorization

Variables	Categories
Individual level factors	
Age of girl at circumcision (years)	(1) 0–4; (2) 5–9; (3) 10–14
Mother’s age (years)	(1) 15–19; (2) 20–24; (3) 25–29; (4) 30–34; (5) 35–49
Marital status	(1) Married; (2) Divorced; (3) widowed; (4) others
Religion	(1) Orthodox; (2) Muslim; (3) Protestant; (4) Others
Mother’s education	(1) No education; (2) Primary; (3) Secondary or higher
Father’s education	(1) No education; (2)Primary; (3)Secondary or higher
Mother’s occupation	(1) Did not work; (2) Non-Professional; (3) Professional
Father’s occupation	(1) Did not work; (2) Non-Professional; (3) Professional
Maternal circumcision status	(1) Circumcised; (2) not circumcised
Wealth index	(1) Poorest; (2)Poorer; (3) Rich; (4) Richer; (5)Richest
Sex of household head	(1) Male; (2) Female
Number of living children	(1) 1–2; (2) 3–4; (3) 5–6; (4) 7+
Mother’s relation to the head of the household	(1) Wife; (2) head; (3) Daughter; (4) Others
Had regular media exposure	(0) No; (1) Yes
Perceived religious believes to FGM/C	(1) Required; (2) Not required; (3) No religion; (4) Don’t know
Women’s opinions towards the practice of FGM/C	(2) Should be continued; (2) should not be continued; (3) Don’t know/depends
Information on FGM/C	(1) Ever heard; (2) Not ever heard
Community level factors	
Residence	(1) Urban; (2) Rural
Region	(1) Somali; (2) Tigray; (3) Afar; (4) Amhara; (5) Oromiya; (6) Benishangul-Gumuz; (7) SNNPR; (8) Gambella; (9) Harari; (10) Addis Ababa (11) Dire Dawa
Ethnicity	(1)Affar; (2) Somali; (3) Amhara; (3) Oromo; (4) Tigray; (5) Sidama; (6) Wolaita; (7) Guragie; (8) Siltie; (9) Dawuro; (10) Kembata; (11) Berta (12) Agew Awi; (13) Hadiya; (14) Others
Proportion of community with regular media exposure	(1) Low; (2) High
Women’s literacy rate of the community	(1) Low; (2) High
Proportion of community in the poor wealth quantiles	(1) Low; (2) High

### Operational definitions

**Circumcision status of girls**: In the 2016 EDHS, women who had daughters were asked if any of their daughters born in 1992 or later had been circumcised. If a girl has been circumcised, she was classified as “Yes”, otherwise “No” [10].

**Mass media exposure**: In the 2016 EDHS, women were asked how often they read a newspaper, listened to the radio, or watched television. Those who responded at least once a week are considered to be regularly exposed to that form of media [10].

**Proportion of community with regular media exposure**: The community level media exposure was categorized as “high” if the proportion of the communities’ regular exposure to mass media is above the national median value, otherwise “low”. The proportion was computed by aggregating individual’s regular media exposure in cluster.

**Proportion of community in the poor wealth quantile:** The community level poor wealth index was categorized as “high” if the proportion of the communities in the poor wealth quantile is above the national median value, otherwise “low”. The proportion was computed by aggregating individual’s wealth index in cluster.

### Data management and statistical analysis

Further recoding of variables was done to better suit with other studies for comparison. Data were analyzed using Stata Version 14 [21] for the non-spatial statistical analysis, and SaTScan v9.6 (https://www.satscan.org) [26] as well as ArcGIS 10.5 (http://www.esri.com) for the spatial analysis. Sampling weights were applied to ensure sample representativeness, and the detailed weighting procedure is found in the EDHS final report [10].

**Spatial analysis:** To evaluate whether the pattern of FGM/C is clustered, dispersed or random across the whole study area, global spatial autocorrelation was assessed using a global statistic called Moran’s I. A positive and statistically significant Moran’s I value (positive spatial autocorrelation) indicates the clustering of like values, while a negative and significant Moran’s I value (negative spatial autocorrelation) indicates a dispersed pattern [27]. As a positive spatial autocorrelation was explored, Kulldorff’s method of Poisson-based purely spatial scan statistic was employed to detect the local clusters of areas with high rates of FGM/C using SaTScan 9.6 software [26]. “SaTScan™ is a trademark of Martin Kulldorff. The SaTScan™ software was developed under the joint auspices of (i) Martin Kulldorff, (ii) the National Cancer Institute, and (iii) Farzad Mostashari of the New York City Department of Health and Mental Hygiene [26, 28, 29]”.

To detect significant clusters of areas with FGM/C among girls, SaTScan uses a circular window of variable size. The maximum spatial cluster size was set to 25% of the population at risk, and *p*-values for cluster detection were calculated using standard Monte Carlo hypothesis testing with 999 Monte Carlo replicates. Statistically significant cluster was declared when the log likelihood ratio (LLR) is greater than the Standard Monte Carlo critical value for 0.05 significance level, or p-value < 0.05 [26, 28]. In addition, hot spot analysis using the Getis-Ord Gi* statistic was carried out to identify FGM/C hotspots.

A spatial interpolation was used to predict the level of FGM/C for the unmeasured locations in the country from the limited number of sample data points using an inverse distance weighted (IDW) technique. The IDW tool uses a method of interpolation that estimates the percentage of FGM/C by averaging the values of sample data points in the neighborhood locations.

**Multilevel logistic regression analysis**: Considering the hierarchical nature of the 2016 EDHS data in which 6985 girls aged 0–14 years were nested within 603 EAs, we fitted multilevel logistic regression models were fitted to identify predictors of FGM/C at individual and community levels. To compare and select the best fit model, four models were fitted: the first model (model I), also called the null model, was fitted without any predictor variable as a base line model, the second model (model II) was fitted with variabels at individual level, the third model (Model III) was fitted with variables at community level, and the final model (model IV) was fitted with both individual and community level variables. Then the models were compared using deviance information criteria (DIC), and the final best fit model (model IV) was selected as the model with the smallest DIC value [30].

To declare statistical significance, adjusted odds ratio (AOR) with 95% confidence interval was used as a measure of association (fixed effect) between the outcome variables and the possible predictor variables. Intra-class correlation coefficient (ICC), proportional change in variance (PCV), and median odds ratio (MOR) were also used as measures of variation (random effects). ICC is a measure of within-cluster variation, the variation between individuals within a cluster and it was calculated as $\mathrm{ICC}=\frac{V_A}{V_A+\frac{\uppi^2}{3}}=\frac{V_A}{V_A+3.29}$, where V_A_ is the estimated variance of clusters. It is also used to evaluate whether or not the community level (level 2) variation is ignorable. The PCV, which is frequently used to quantify the variation explained by a multilevel model, expresses the total variation attributed to individual and community level factors, and it was computed as $\mathrm{PCV}=\frac{{\mathrm{V}}_{\mathrm{A}}-{\mathrm{V}}_{\mathrm{B}}}{{\mathrm{V}}_{\mathrm{A}}}$, where V_A_ is the variance of the null model (model 1), and V_B_ is variance of the model with individual or/and community characteristics [31]. MOR is the median odds ratio between the individual at higher risk of the outcome and the individual at lower risk of the outcome when two individuals from two different randomly selected clusters are compared (difference in risk are entirely quantified by the cluster-specific random effects). It was computed using the formula: $\mathrm{MOR}=\exp \left(\sqrt{2\ \mathrm{x}\ {\mathrm{V}}_{\mathrm{A}}}\ \mathrm{x}\ 0.6745\right)\approx \exp \left(0.95\sqrt{{\mathrm{V}}_{\mathrm{A}}}\right)$, where V_A_ is the cluster level variance [31, 32].

### Ethical consideration

Ethical approval was obtained from the ethical review committee of the school of Public Health of the Bahir Dar University. In addition, permission was obtained from the DHS program. The Shape files for the administrative boundaries of Ethiopia were downloaded from the DHS program spatial data repository website [https://www.spatialdata.DHSprogram.com]. The detailed ethical issue is found in the final report of the 2016 EDHS [10].

## Results

### Characteristics of study participants and daughters’ circumcision

Nearly three in seven (44%) women were within the age group of 35–49 years with the mean age of 22.9 years (SD ± 7.0), and over 90% of them were married. Over 70% of mothers of the girls had no education, about one-fifth (20%) of them were in the poorest wealth quintile. Nearly four-fifth (82%) mothers of the daughters had no regular media exposure. Four in five (80%) mothers of the daughters had a history of circumcision, and about 3 in 10 women (30%) believed that FGM/C is required by their religion. Twenty-three percent of women believed that the practice should be continued. Nearly one-fourth (24%) of daughters of the women aged 35–49 years, and more than one-fifth (21%) of the daughters of women with no education were circumcised. Overall, 18.6% of daughters were experienced FGM/C (Table 2).

**Table 2:** Characteristics of study participants and the distribution of daughters’ FGM/C by background characteristics in Ethiopia, 2016

Background Characteristics	Total number of daughters		Daughters circumcised	
	Frequency	Percent	Frequency	Percent
Maternal age (in years) (mean = 22.9, SD + 7.04)				
15–19	84	1.2	4	4.8
20–24	521	7.5	59	11.3
25–29	1486	21.3	196	13.2
30–34	1818	26.0	305	16.8
35–49	3077	44.0	739	24.0
Mother’s marital status				
Married	6386	91.4	1185	18.6
Divorced	245	3.5	61	24.9
Widowed	205	2.9	37	18.0
Others*	149	2.1	19	12.8
Religion				
Orthodox Christian	2483	35.5	655	26.4
Muslim	2677	38.3	382	14.3
Protestant	1654	23.7	255	15.4
Others**	171	2.5	11	6.4
Mother’s educational status				
No education	4952	70.9	1040	21.0
Primary	1642	23.5	253	15.4
Secondary and above	391	5.6	9	2.3
Husband/partner’s education				
No education	4952	70.9	1040	21
Primary	1642	23.5	253	15.4
Secondary and above	391	5.6	9	2.3
Mother’s occupation				
Do not work	3716	53.2	662	17.8
Non-professional	3174	45.4	638	20.1
Professional	96	1.4	2	2.1
Husband/partner’s occupation				
Do not work	499	7.7	111	22.2
Non-professional	5685	88	1041	18.3
Professional	278	4.3	44	15.8
Maternal circumcision status				
Not circumcised	1383	19.8	60	4.3
Circumcised	5602	80.2	1243	22.2
Wealth Index				
Poorest	1429	20.5	284	19.9
Poorer	1445	20.7	276	19.1
Middle	1477	21.1	284	19.2
Richer	1541	22.1	328	21.3
Richest	1093	15.6	130	11.9
Number of living children				
1–2	1152	16.5	132	11.5
3–4	2094	30	369	17.6
5–6	2080	29.8	463	22.3
7+	1659	23.8	338	20.4
Sex of household head				
Male	5765	82.5	1036	18
Female	1220	17.5	267	21.9
Mother’s relation to the head of the household				
Wife	5535	79.2	1014	18.3
Head	1045	15	236	22.6
Daughter or daughter-in-law	307	4.4	35	11.4
Other***	98	1.4	17	17.3
Had regular media exposure				
No	5706	81.7	1202	21.1
Yes	1279	18.3	101	7.9
Perceived religious believes to FGM/C				
Not required	4531	64.9	645	14.2
Required	2085	29.8	580	27.8
Do not know/ no religion	369	5.3	77	20.9
Opinions of women towards the practice of FGM/C				
Stop	5058	72.4	692	13.7
Continue	1593	22.8	557	35
Depends/ do not know	334	4.8	53	15.9
Ever heard of female circumcision				
No	88	1.3	4	4.5
Yes	6897	98.7	1298	18.8
Total	**6985**	**100**	**1302**	**18.6**

*Catholic, traditional, and other; ** never in union, separated/no longer living together; *** Daughter; granddaughter; mother, sister, other relative, adopted/foster child and not related

**Fig. 1:**
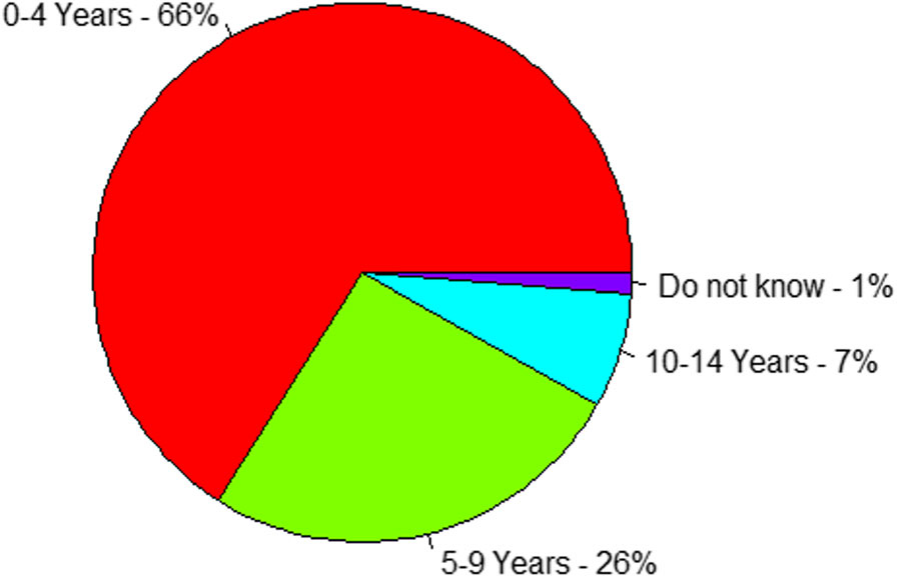
Distribution of girls by age at circumcision, EDHS 2016

Most of daughters (66%) were circumcised within the ages of 0 to 4 years, whereas, nearly the one-fourth (26%) of the daughters were circumcised at the ages of 5 to 9 years (Fig. 1).

### Community level characteristics

The majority (89%) were study participants were rural residents. Nearly two-fifth (40%) and one-fourth (25%) of the study participants were Oromo and Amhara in their Ethnicity, respectively. Nearly one-third (67%) of women’s literacy rate was low, and almost half (50%) of the communities had low regular media exposure. The proportion of community in the poor wealth quantile was 75% (Table 3**).**

**Table 3:** Community level characteristics and distribution of daughters’ circumcision by community level characteristics in Ethiopia, 2016

Background characteristics	Total number of daughters		Daughters circumcised	
	Frequency	Percent	Frequency	Percent
Residence				
Urban	794	11.4	70	8.8
Rural	6191	88.6	1233	19.9
Region				
Tigray	390	5.6	52	13.3
Afar	65	0.9	51	78.5
Amhara	1473	21.1	581	39.4
Oromia	3019	43.2	269	8.9
Somali	238	3.4	76	31.9
Benishangul Gumuz	72	1.0	17	23.3
SNNPR	1532	21.9	247	16.1
Gambella	10	0.1	1	10.0
Harari	17	0.2	2	11.8
Addis Ababa	140	2.0	2	1.4
Dire Dawa	29	0.4	4	13.8
Ethnicity				
Affar	60	0.9	49	81.7
Somalie	227	3.2	71	31.3
Amhara	1749	25.0	547	31.3
Oromo	2812	40.3	302	10.7
Tigrie	394	5.6	51	12.9
Sidama	340	4.9	49	14.4
Wolaita	228	3.3	77	33.8
Guragie	115	1.6	16	13.9
Siltie	109	1.6	13	11.9
Dawuro	62	0.9	27	42.9
Kembata	61	0.9	12	19.7
Berta	25	0.4	12	48.0
Agew Awi	11	0.2	5	45.5
Hadiya	218	3.1	42	19.4
Others*	574	8.2	29	5.0
Women’s literacy rate of the community				
Low	4746	67.9	880	18.6
High	2239	32.1	422	18.7
Proportion of community with regular media exposure				
Low	3465	49.6	791	22.8
High	3520	50.4	511	14.5
Proportion of community in the poor wealth quantile				
Low	1735	24.8	359	20.6
High	5250	75.2	943	17.9
Total	**6985**	**100.0**	**1302**	**18.6**

*Nuwer, Kefficho, Gumuz, Gamo, Anyiwak, Gedeoi, Derashe, Dizi, Agew Hamyra, Goffa, Argoba, Ari, Harari, Basketo, Konta, Komo, Koyego, Mao, Bench, Shekecho, Sheko, Shinasha, Burji, Bena, Yem, etc.

### Spatial pattern of FGM/C

**Fig. 2:**
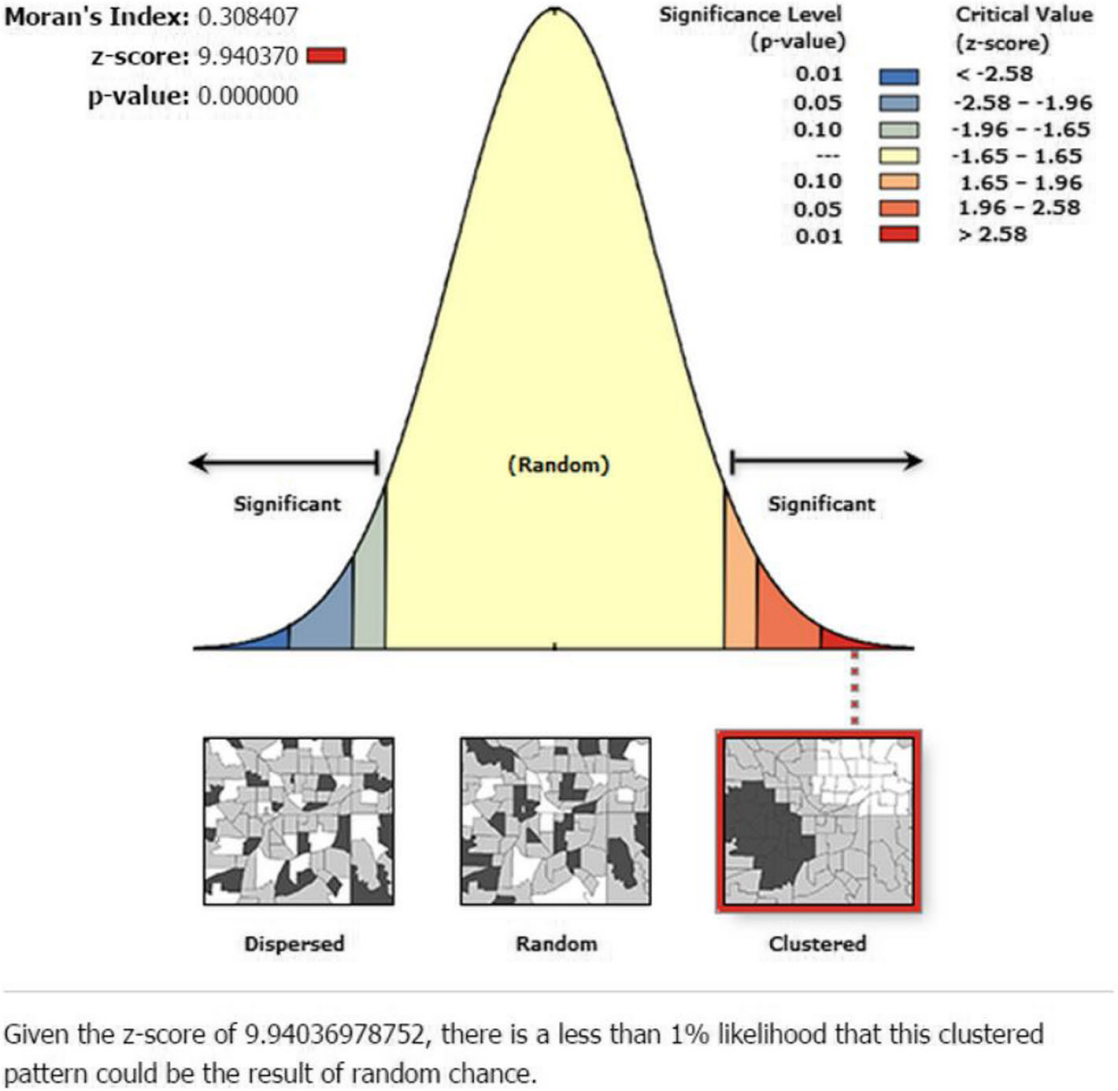
The global spatial autocorrelation analysis result to evaluate the spatial pattern of FGM/C among girls in Ethiopia, 2016

The global test for spatial autocorrelation revealed that, a clustered pattern of daughters’ FGM/C was observed across the study areas (Moran’s I = 0.31, *p*-value < 0.001) (Fig. 2).

**Fig. 3:**
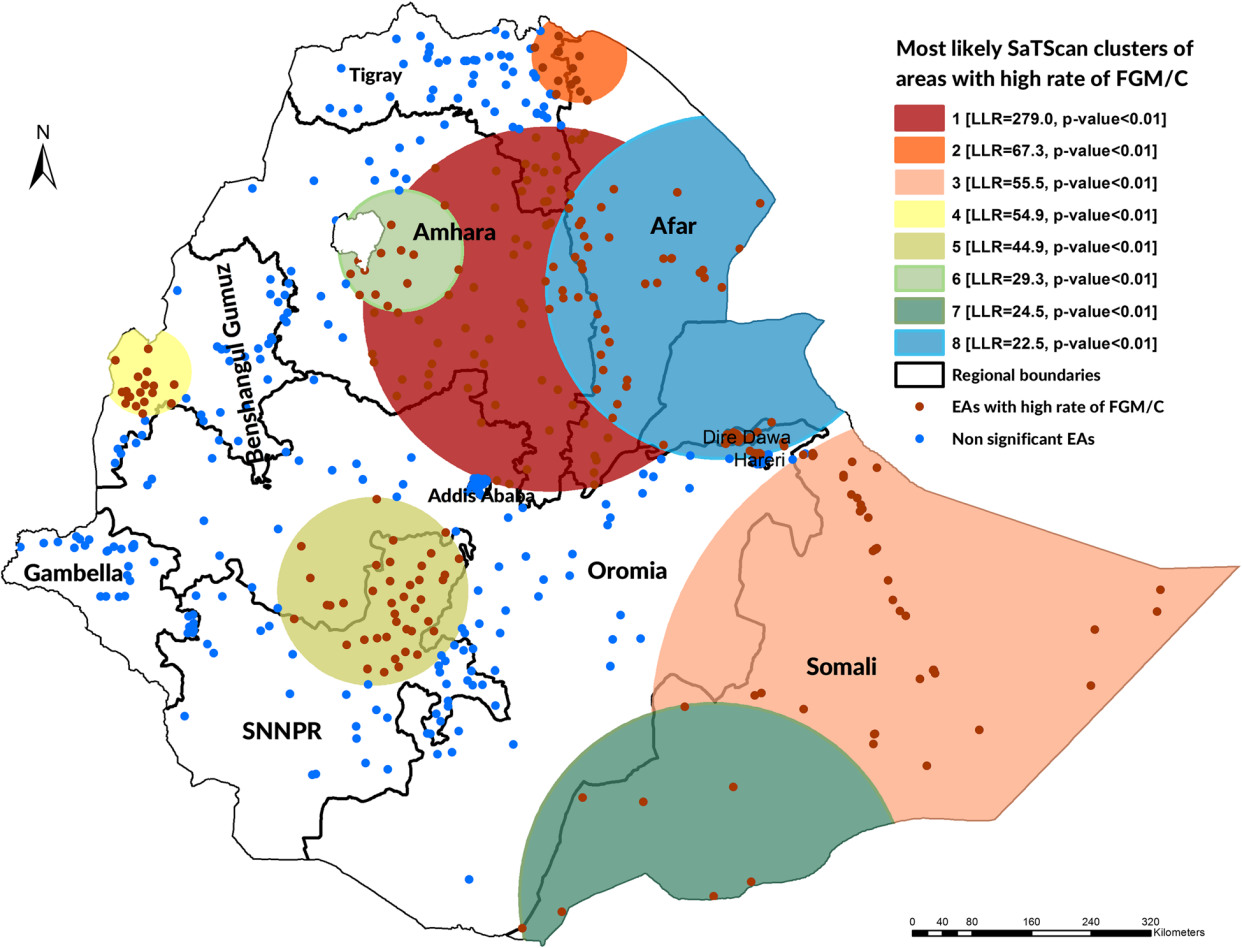
Spatial clustering of areas with high rate of daughters’ FGM/C in Ethiopia, 2016

The most likely primary SaTScan cluster of areas with high rate of FGM/C was detected in Afar, Amhara, Tigray, and Oromia regions (LLR = 279.0, *p* < 0.01). The secondary SaTScan cluster was also detected in Tigray, and Afar regions (LLR = 67.3, p < 0.01). Somali (LLR = 55.5, *P* < 0.01), Benishangul Gumuz (LLR = 54.9, P < 0.01), Oromia (LLR = 44.9, P < 0.01), and Amhara (LLR = 29.3, P < 0.01) were the regions in which the third, fourth, fifth, and sixth most likely clusters of areas with high rate of daughters’ FGM/C were detected, respectively. In addition, the seventh most likely SaTScan cluster was also detected in Somali region (LLR = 24.5, P < 0.01), while the eighth most likely SaTScan cluster (LLR = 22.5, P < 0.01) in Afar, Oromia and Amhara regions, including Dire Dawa Town (Table 4**and** Fig. 3).

**Table 4:** Spatial clusters female genital mutilation/cutting among girls in Ethiopia, 2016

Cluster	Regions	Radius (km)	Obs.*	Exp.**	RR	LLR	p-value
1	Amhara, Afar, Tigray, and Oromia regions	243.6	682	279.7	3.60	279.0	0.001
2	Tigray and Afar regions	60.1	95	22.6	4.61	67.3	0.001
3	Somali region	437.5	200	92.9	2.57	55.5	0.001
4	Benishangul Gumuz region	57.1	88	23.8	4.21	54.9	0.001
5	Oromia and SNNPR regions	124.9	140	61.5	2.83	44.9	0.001
6	Amhara region	80.8	28	4.4	6.91	29.3	0.001
7	Somali region	259.5	29	5.8	5.46	24.5	0.001
8	Afar, Oromia, and Amhara regions including Dire Dawa Town	230.7	84	40.4	2.59	22.5	0.001

RR: Relative risk; LLR: Log likelihood ratio; *Number of observed cases in a cluster; **Number of expected cases in a cluster

**Fig. 4:**
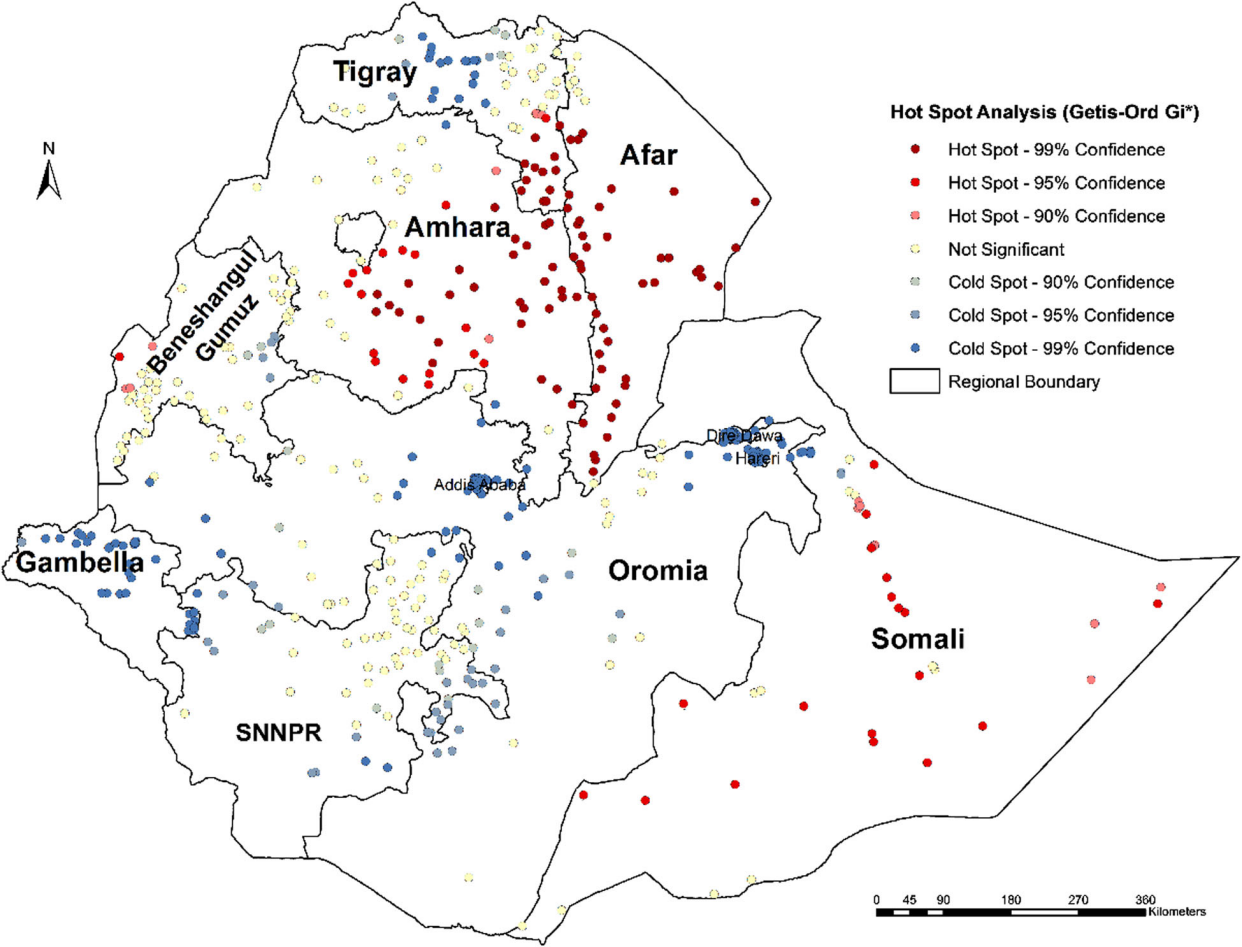
FGM/C hotspots in Ethiopia (Getis-Ord-Gi* statistic), EDHS 2016

In addition, the Getis-Ord Gi* statistic identified significant hotspots and cold spots of female genital mutilation/cutting which are almost the same as the clusters detected by the Kulldorff’s scan statistic (Figs. 3 and 4).

**Fig. 5:**
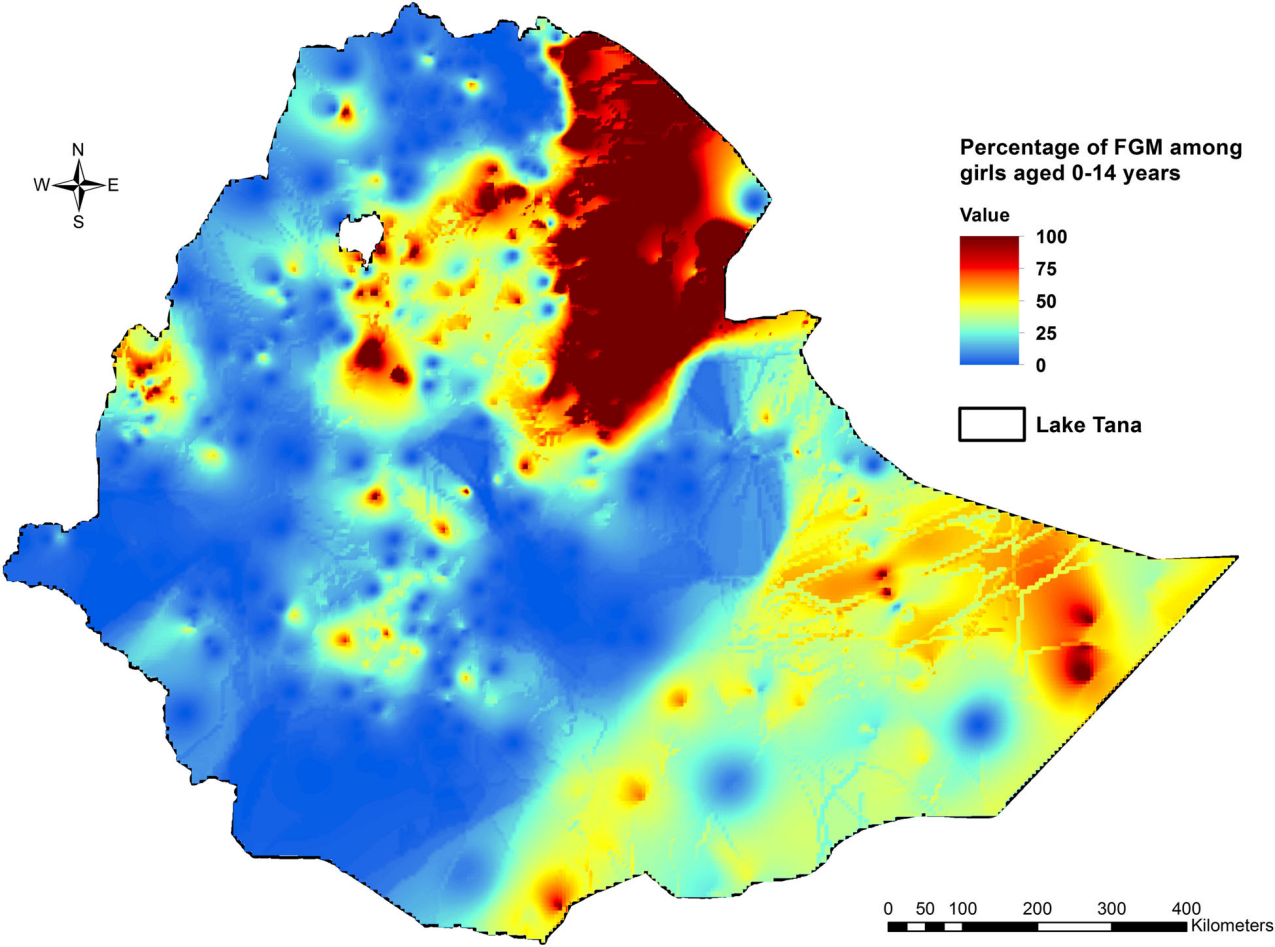
A spatial interpolation of FGM/C among girls in Ethiopia from the sample data points using an inverse distance weighted (IDW) technique.

Figure 5 shows the spatial interpolation of FGM/C using inverse distance weighted (IDW) method, which predicts the percentage of FGM/C among girls for the unmeasured location across the country from the limited number of sample data points. More than 75% of the girls were circumcised in most part of Afar region (Fig. 5).

### Multilevel analysis

In the multilevel regression analysis where both individual and community level factors were included, the final best fit model (model IV) revealed that maternal age, maternal education, households wealth index, maternal circumcision status, number of living children, regular exposure to media, perceived religious believes to FGM/C, women’s opinion towards the practice of FGM/C were statistical significant individual-level factors associated with the daughters’ FGM/C. In addition, place of residence, geographical region, ethnicity, and community with regular media exposure were the community level factors associated with the practice of FGM/C (Table 5).

**Table 5:** Individual and community level factors affecting daughters’ FGM/C in Ethiopia, 2016 (a multilevel logistic regression analysis)

Variables	Model II AOR (95%CI)	Model IIIAOR (95%CI)	Model IVAOR (95%CI)
**Individual level factors**			
Maternal age (in years)			
15–19	1.00	–	1.00
20–34	1.19 (0.53–2.7)	–	1.61 (0.68–3.8)
35–49	2.31 (1.00–5.36)	–	3.51 (1.45–8.5)*
Religion			
Orthodox Christian	1.00	–	1.00
Muslim	1.52 (1.03–2.24)	–	1.05 (0.7–1.58)
Protestant	0.67 (0.42–1.07)	–	0.89 (0.54–1.47)
Others*	0.23 (0.05–0.94)	–	0.29 (0.08–1.03)
Mother’s educational status			
No education	1.00	–	1.00
Primary	0.96 (0.76–1.22)	–	1.06 (0.84–1.34)
Secondary and above	0.29 (0.15–0.58)	–	0.43 (0.22–0.84)*
Mother’s occupation			
Do not work	1.00	–	1.00
Non-professional	1.04 (0.85–1.27)	–	1.08 (0.88–1.31)
Professional	0.44 (0.13–1.46)	–	0.51 (0.16–1.66)
Maternal circumcision status			
Not circumcised	1.00	–	1.00
Circumcised	7.37 (4.69–11.59)	–	7.49 (4.84–11.59)*
Wealth Index			
Poorest	1.00	–	1.00
Poorer	0.91 (0.68–1.23)	–	1.25 (0.93–1.67)
Middle	0.89 (0.66–1.22)	–	1.30 (0.96–1.76)
Richer	0.30 (0.10–0.51)	–	0.54 (0.30–0.82)*
Richest	0.65 (0.43–0.89)	–	0.70 (0.46–0.92)*
Number of living children			
1–2	1.00	–	1.00
3–4	1.28 (0.94–1.74)	–	1.27 (0.93–1.72)
5–6	2.03 (1.47–2.8)	–	1.89 (1.37–2.60)*
7+	1.82 (1.28–2.61)	–	1.62 (1.13–2.32)*
Sex of household head			
Male	1.00	–	1.00
Female	1.38 (0.62–3.08)	–	1.41 (0.62–3.22)
Mother’s relation to the head of the household			
Wife	1.00	–	1.00
Head	1.06 (0.46–2.44)	–	1.02 (0.44–2.4)
Daughter or daughter-in-law	0.88 (0.44–1.76)	–	0.81 (0.4–1.67)
Other	0.88 (0.35–2.19)	–	0.71 (0.28–1.8)
Had regular media exposure			
No	1.00	–	1.00
Yes	0.56 (0.39–0.80)	–	0.51 (0.35–0.77)*
Perceived religious believes to FGM/C			
Not required	1.00	–	1.00
Required	1.57 (1.25–1.97)	–	1.47 (1.17–1.83)*
Do not know/ no religion	1.17 (0.74–1.84)	–	1.16 (0.75–1.80)
Opinions of women towards the practice of FGM/C			
Stop	1.00	–	1.00
Continue	2.68 (2.12–3.38)	–	2.50 (1.99–3.14)*
Depends/ do not know	1.46 (0.88–2.42)	–	1.53 (0.93–2.51)
Ever heard of female circumcision			
No	1.00	–	1.00
Yes	1.52 (0.35–6.6)	–	1.67 (0.38–7.42)
**Community level factors**			
Residence			
Urban	–	1.00	1.00
Rural	–	3.29 (1.87–5.78)	2.51 (1.46–4.33)*
Region			
Afar	–	1.00	1.00
Tigray	–	0.04 (0.01–0.19)	0.07 (0.01–0.36)*
Amhara	–	0.60(0.23–1.58)	0.56 (0.21–1.49)
Oromia	–	0.05 (0.02–0.13)	0.05 (0.02–0.13)*
Somali	–	0.35 (0.10–1.17)	0.24 (0.07–0.77)*
Benishangul Gumuz	–	0.10 (0.03–0.28)	0.13 (0.05–0.37)*
SNNPR	–	0.09 (0.03–0.26)	0.09 (0.03–0.27)*
Gambella	–	0.06 (0.02–0.21)	0.13 (0.04–0.44)*
Harari	–	0.07 (0.02–0.21)	0.06 (0.02–0.16)*
Addis Ababa	–	0.03 (0.01–0.10)	0.05 (0.01–0.19)*
Dire Dawa	–	0.16 (0.05–0.45)	0.11 (0.04–0.30)*
Ethnicity			
Afar	–	1.00	1.00
Somalie	–	0.15 (0.05–0.49)	0.18 (0.05–0.56)*
Amhara	–	0.08 (0.03–0.19)	0.18 (0.07–0.45)*
Oromo	–	0.09 (0.04–0.23)	0.15 (0.06–0.38)*
Tigrie	–	0.16 (0.04–0.65)	0.43 (0.09–2.16)
Sidama	–	0.09 (0.03–0.34)	0.16 (0.04–0.61)*
Wolaita	–	0.40 (0.13–1.24)	0.70 (0.21–2.29)
Guragie	–	0.14 (0.04–0.44)	0.19 (0.06–0.62)*
Siltie	–	0.06 (0.01–0.30)	0.11 (0.02–0.57)*
Dawuro	–	0.81 (0.10–6.84)	1.38 (0.21–9.24)
Kembata	–	0.30 (0.07–1.35)	0.54 (0.12–2.54)
Berta	–	0.67 (0.19–2.36)	0.77 (0.23–2.61)
Agew Awi	–	0.03 (0.00–0.26)	0.14 (0.02–1.27)
Hadiya	–	0.16 (0.05–0.54)	0.29 (0.08–1.01)
Others	–	0.04 (0.02–0.12)	0.11 (0.04–0.31)*
Women’s literacy rate of the community			
Low	–	1.00	1.00
High	–	0.84 (0.55–1.28)	0.88 (0.60–1.28)
Proportion of community with regular media exposure			
Low	–	1.00	1.00
High	–	0.67 (0.42–0.88)	0.70 (0.48–0.91)*
Proportion of community in the poor wealth quantile			
Low	–	1.00	1.00
High	–	0.71 (0.44–1.14)	0.79 (0.51–1.21)

*p-value< 0.05

**Individual level factors**: The daughters of women aged 35–49 years were 3.5 times (AOR = 3.51; 95% CI: 1.45–8.5) more likely to be circumcised than the daughters of women aged 15–19 years. Daughters whose maternal educational status was secondary and above were 57% less likely to experience genital mutilation (AOR = 0.43; 95% CI: 0.22–0.84) compared to those daughters with no maternal education. The odds of daughters’ circumcision were decreased by 57% (AOR = 0.43; 95% CI: 0.22–0.84) in daughters with secondary and higher maternal education compared to daughters whose mothers had no education. The daughters of circumcised women were seven times more likely (AOR = 7.49; 95% CI: 4.84–11.59) to be circumcised as compared with their counter parts.

The odds of daughters circumcision in the households with richer and richest wealth quantile were 0.46 times (AOR = 0.54; 95% CI: 0.30–0.82), and 0.7 times (AOR = 0.70; 95% CI 0.46–0.92) higher than those daughters from the households with the poorest wealth quantile, respectively. The odds of circumcision among daughters from the households having 5 to 6 and seven or more number of living children were 1.89 times (AOR = 1.89, 95% CI 1.37–2.60), and 1.62 times (AOR =1.62; 95% CI 1.13–2.32) higher than those daughters from the households having two or less number of living children, respectively. The daughters of women who believe that FGM/C is required by their religion were more likely (AOR = 1.42, 95% CI: 1.17–1.83) to have circumcision than their counter parts. The odds of circumcision among daughters from women who support the continuation of FGM/C practice was 2.5 times more likely (AOR = 2.50, 95% CI: 1.99–3.14) than the daughters from the women who did not support the continuation of the practice. Women’s regular media exposure decreases the odds of circumcision in their daughters by 49% (AOR = 0.51; 95%CI: 0.35–0.77) compared to their counter parts (Table 5).

**Community level factors:** The odds of daughters’ FGM/C were increased by 51% (AOR =2.51; 95% CI: 1.46–4.33) among daughters living in rural areas compared to the daughters living in urban areas. Compared with Afar region, the odds of daughters’ FGM/C were lower in Tigray (AOR =0.07; 95% CI: 0.01–0.36), Oromia (AOR =0.05; 95% CI: 0.02–0.13), Somali (AOR =0.24; 95% CI: 0.07–0.77), Benishangul Gumuz (AOR = 0.13; 95%CI: 0.05–0.37), SNNPR (AOR = 0.09; 95%CI: 0.03–0.27), Gambella (AOR = 0.13; 95%CI: 0.04–0.44) regions including Harari, Addis Ababa, and Dire Dawa towns. In addition, the practice of FGM/C is less likely in ethnicities of Somali, Amhara, Oromo, Sidama Guragie, and Siltie compared with Afar Ethnicity. The higher proportion of community with regular media exposure decreases the odds of circumcision in their daughters by 30% (AOR = 0.70; 95% CI: 0.48–0.91) compared to their counterparts (Table 5).

### Measures of variation (random-effects) and model fit statistic

As the multilevel logistic regression analysis results depicted in Table 6, the null model revealed statistically significant variation in FGM/C among communities [τ = 5.86, *p* < 0.001], and the odds of FGM/C in daughters was attributed to community-level factors (ICC =64%). After incorporating the individual level characteristics to the null model, the variation in the odds of FGM/C among the communities remained significant [τ = 3.19, p < 0.001] with the ICC of 49%. As compared to the variance in model 2, incorporating community-level characteristics to the null model decreased the variance in FGM/C among the communities [τ = 1.58, p < 0.001], and also the ICC lowered to 32.4%.

**Table 6:** Results from random intercept model (measures of variation) for daughters’ circumcision at cluster level by multilevel logistic regression analysis

Measures of variation	Model 1^a^	Model 2^b^	Model 3^c^	Model 4^d^
Community level				
Variance (SE)	5.863 (0.129)*	3.189 (0.103)*	1.577 (0.084)*	0.988 (0.076)*
Explained variation (PCV)	Reference	45.6	73.1	83.1
ICC (%)	64.1	49.2	32.4	23.1
MOR^e^	9.98	5.45	3.30	2.57
Model fit statistics				
DIC (−2log likelihood)	5197	4626	4736	4272

*p-value < 0.001

SE: Standard Error; PCV: Proportional Change in Variance; ICC: Intraclass Correlation Coefficient; MOR: Median Odds Ratio; DIC: Deviance Information Criterion.^a^Model 1 is the null model, a baseline model without any predictor variable^b^Model 2 is the model fitted with individual level factors^c^Model 3 is the model fitted with community level factors^d^Model 4 is the final model fitted with both individual and community level factors

^e^Increased risk (in median) that one would have if moving to a neighborhood/cluster with a higher risk

The final best fit model (model 4) which incorporated both the individual and community level characteristics revealed statistically significant variability in the odds of FGM/C among the communities [τ = 0.99, p < 0.001] with the ICC of 23%. The PCV comparing the null model (Model 1) with the model containing both individual and community characteristics (Model 4) was 0.831, and it indicated that about 83% of the variance in the odds of FGM/C was attributed to both individual and community-level characteristics.

In this study, the MOR was 9.98 in the null model (model 1), 5.45 in model 2, 3.30 in model 3, and 2.57 in model 4. Hence, MOR of 9.98 in the null model indicated the presence of heterogeneity across communities, and incorporating both individual and community level factors to the null model could reduce the unexplained heterogeneity to the MOR of 2.57 (Table 6).

## Discussions

In this study, the overall prevalence of FGM/C among girls was found to be 18.6%, which indicates declining as compared to the national prevalence (24%) reported in the 2005 EDHS [9]. The finding is supported by other evidences which revealed that Ethiopia has made significant progress in the last two decades in reducing girls’ vulnerability to FGM/C [9, 10, 13]. It is also low as compared to the most recent DHS reports of other African countries such as Mali, 73%, and Guinea, 39% [33]. Despite this progress, many girls are still at risk with geographical variation [13]. On the other hand, the practice is high as compared with similar survey reports of Egypt, 12% [34], Kenya, 3% [35], chad, 10% [33, 36]%, Tanzania, 0.4% [37].

The global test for spatial autocorrelation indicated the presence of clustering in FGM/C across the study areas without specifying the spatial location (Moran’s I = 0.31, *p*-value< 0.01). This could indicate that the practice of FGM/C is clustered across the communities in the country, and suggested an extended local test, a focused test which used to detect local clusters of areas with high rate of FGM/C. The results of other studies in and outside Ethiopia also supported this finding of spatial clustering in FGM/C practice [24, 38].

The Kulldorff’s spatial scan statistic detected FGM/C ‘hotspots’ among girls in Ethiopia. Moreover, the results of the Getis-Ord Gi* statistic supported and confirmed the results of the findings of the Kulldorff’s purely spatial analysis.

In this study, northeastern parts of the country, particularly in Afar, Amhara, Tigray and Oromia regions, were detected as FGM/C hotspots. This may be due to the fact that the neighborhood areas of the regions may share common characteristics in relation to traditional practices. In addition, FGM/C hotspots were detected Somali, Benishangul Gumuz, and SNNPR regions including Dire Dawa Town. This finding can indicate that the communities located in the Northern, Northeastern and Eastern parts of the country are FGM/C ‘hotspots’ and are areas in need of urgent attention. This local clustering of FGM/C can also indicate that daughters who lived in the FGM/C hotspot areas had a high probability of experiencing circumcision compared with those who lived outside the identified clusters. This may be due to differences in community background characteristics such as Ethnicity, religion, community perception to FGM/C, and this identification of clusters with high FGM/C practice can lead to targeted interventions.

The multilevel regression analysis confirmed that the variation in daughters’ FGM/C in Ethiopia was attributed to both individual and community level factors which accounted for 83% of the variation.

Like other studies [24, 39], this study identified maternal age, household wealth index, maternal education, number of living children, perceived religious believes to FGM/C, opinions of women towards the practice of FGM/C, and regular media exposure as individual level factors associated with the practice of FGM/C.

In this study, older maternal age was found to be a factor to increase the risk of FGM/C among their daughters. This could be justified by lower awareness of FGM/C impacts in older age women, and older women may be more likely to conform to traditional practices, including FGM/C.

The results of this study showed that the daughters of women with secondary or higher educational status had lower odds of experiencing FGM/C compared to the those who had no education, and it is supported by other studies conducted in and out of Ethiopia [24, 39]. This less risk of daughters for FGM/C in educated mothers could be justified as women who had completed secondary school or above are more likely to be aware of the FGM/C risks; they may have better opportunity in information communication, and lower tendency to practice FGM/C on their daughters. In addition, educated women are more likely to evaluate their own beliefs and values related to the practice [1]. Moreover, education empowers women not only to get new knowledge but also improve the sharing of experiences [1].

Compared to the daughters of uncircumcised women, the daughters of circumcised women are at higher risk of FGM/C, and this finding is consistent with another study finding in Kenya [39]. This may be due to the higher tendency of circumcised women to support the continuation of FGM/C than the uncircumcised women. This finding may suggest that communities who are currently practicing FGM/C among the daughters may also be at considerable risk of continuing the FGM/C practice persistently in the future.

Like what has been documented elsewhere [24, 40–42], this finding of this study revealed that the risk of FGM/C among girls is lower in the households with better wealth status compared to their counterparts. The possible reason for this could be due to the decision making power of women in relation to their wealth. As evidences revealed, improved economic status can enhance the role of women in decision making not to support the continuation of FGM/C practice [38, 43, 44].

In many communities, the practice of FGM/C is believed to be a deeply rooted traditional practice, which is attributed to the cultures and religion [1, 2, 7, 39, 45]. However, the finding of this study could not show statistically significant association between religion and the practice of FGM/C. This finding also contradicted with the findings of other studies in Ethiopia, and Kenya [24, 39], where a statistical association between religion and the practice FGM/C was reported. Other evidences indicated that the practice of FGM/C is more likely in Muslim communities [39, 46]. However, not only Muslim communities practice FGM/C, but also many other non-Muslim ones do [39]. This can suggest that the association between the practice of FGM/C and religion varies widely across countries, regions and communities. On the other hand, this study revealed that the daughters of women who believed as FGM/C is required by religion were more likely to be circumcised as compared to their counter parts. The daughters were less likely to be circumcised if their mothers had regular mass media exposure, which is supported by the findings of another studies [24, 44].

Among the community level characteristics incorporated in this study, place of residence, geographical region, ethnicity and proportion of community with regular media exposure were identified as factors affecting the practice of FGM/C. In contrast to a study findings in Kenya [38], this study revealed that the daughters in the rural areas are at higher risk of FGM/C than the daughters in urban areas. This could be justified in that communities in rural area may be more likely to have limited cultural diversity, and in communities where continuation of FGM/C is supported, it could be challenging to escape conventions or norms that hold the practice in place [39]. On the other hand, urban areas may be more in cultural diversity, and communities may have better opportunity to learn from setting such as school, church, work, home, or others [39]. Daughters from Tigray, Oromia, Somali, Benishangul Gumuz, SNNPR, and Gambella regions as well as Harari, Addis Ababa, and Dire Dawa towns had lower risk of FGM/C compared to the daughters in Afar region. A previous study conducted in Ethiopia supported this geographical disparity in daughters’ FGM/C [24]. This may be due to the socio-cultural differences across the regions of the country such as ethnicity, economic development, and women’s education.

As evidences revealed, the practice of FGM/C is associated with ethnic identity [1, 7, 39]. This study also found a significant association between ethnicity and the practice of FGM/C. As the findings of this study revealed, the girls in the Ethnicities of Somalie, Amhara, Oromo, Sidama Guragie, and Siltie had lower risk of having FGM/C as compared to the girls in the ethnic group of Afar. This could be explained in that ethnicity may be a proxy for shared norms related to marriageability, sexual restraint, or other shared values [39]. Hence, this may suggest a call for targeted intervention programs to reduce or eliminate FGM/C.

This study also identified that higher proportion of the community with regular media exposure lowers the risk of FGM/C in daughters by 30% as compared to the lower proportion of the community with regular media exposure. This may be due to the fact that media exposure has an important role in disseminating health information; and communities who have media exposure are more likely to have information on the risks of FGM/C practice.

In this study, the results should be interpreted cautiously since it requires an understanding that not all girls have reached their final cutting status; some of the girls who are not cut yet may still be cut in the future. Applying both spatial and non-spatial statistical methods for a more comprehensive and sound analysis was the strength of this study. However, girls from the EAs with no the geographical coordinates were not included in in this analysis and this may affect the results of the study including the generalizability. Hence, it could be mentioned as the limitation of the study.

## Conclusion

In conclusion, the spatial distribution of FGM/C among girls in Ethiopia exhibits a clustered pattern. FGM/C hotspots were detected in Afar, Amhara, Tigray, Benishangul Gumuz, Oromia, SNNPR and Somali regions including Dire Dawa Town. Both individual and community level factors play a significant role in the practice of FGM/C among girls, which accounted for 83% variation across the communities. Older maternal age, lack of maternal education, maternal circumcision, better wealth index, higher number of living children, regular media exposure, perceived religious believes to FGM/C, women’s opinion towards the practice of FGM/C were statistical significant individual-level factors associated with the daughters’ FGM/C. Whereas, place of residence, geographical region, ethnicity, and community media exposure were the community level factors associated with the practice of FGM/C among daughters. Hence, it is good if the federal ministry of health of Ethiopia and other concerned reproductive health programmers give priority for the FGM/C hotspots identified in this study. Furthermore, elimination of FGM/C requires integrated interventions both at individual and community levels: improving women’s education, promoting media utilization, education of women on religious believes to FGM/C, and changing their attitudes towards the practice of FGM/C, and improving the households’ wealth status.

## Data Availability

The datasets used for this are available in the DHS program repository, (https://www.dhsprogram.com/data/dataset_admin) to all registered users.

## Abbreviations

AOR: adjusted odds ratio
CI: confidence intervals
DHS: demographic and health survey
DIC: Deviance information criterion
EAs: Enumeration areas
EDHS: Ethiopian demographic and health survey
FGM/C: Female genital mutilation/cutting
GIS: geographic information system
ICC: intracluster correlation
LLR: Log likelihood ratio
MOR: median odds ratio
PCV: proportional change in variance
PHC: population and housing census
SD: standard deviation
SNNPR: Southern Nations, Nationalities, and People Region
WHO: world health organization
